# [18F]-T807 tauopathy PET imaging in chronic traumatic encephalopathy

**DOI:** 10.12688/f1000research.5372.1

**Published:** 2014-09-29

**Authors:** Sam Gandy, Steven T. DeKosky

**Affiliations:** 1 Icahn School of Medicine at Mount Sinai, New York, 10029, USA; 2 University of Virginia School of Medicine, Charlottesville, 22908, USA

## Abstract

A new molecular ligand for positron emission tomography (PET) of the human brain, [18F]-T807, is under investigation for the antemortem detection of pathological neurofibrillary aggregates, which are evidence of neurofibrillary tangle (NFT) diseases, also known as tauopathies. Repetitive mild traumatic brain injuries in athletes and battlefield veterans are associated with one such tauopathy, known as chronic traumatic encephalopathy (CTE). In a recent case report, a former NFL player with clinically probable CTE and a concurrent Progressive Supranuclear Palsy (PSP) –like syndrome was studied using [18F]-T807. The interpretation of this player’s [18F]-T807 PET imaging was complicated by the overlap of tracer uptake in brain regions involved in CTE and PSP with regions associated with either nonspecific [18F]-T807 ligand binding or “aging-associated” binding of [18F]-T807 to authentic tauopathy known to be associated with aging and disease severity (i.e., NFT in the mesial temporal lobe). The implications of these data for the utility of [18F]-T807 in the pre-mortem detection of CTE are summarized.

## Correspondence

We recently reported the case of a retired NFL player with a clinical diagnosis of chronic traumatic encephalopathy (CTE); the diagnosis was based on clinical characteristics of his progressive cognitive disorder and negative [18F]-florbetapir PET brain imaging indicating absence of amyloid plaques in his brain (Mitsis *et al.* 2014) ^1^. An [18F]-T807 PET imaging study revealed retention of [18F]-T807 in the substantia nigra (SN), globus pallidi (GP), and hippocampi, bilaterally. Key images from that paper were reproduced in a recent review by Gandy *et al.* (2014) ^2^. The pattern of retention was interpreted by the original authors as potentially consistent with the clinical diagnosis of CTE ^1^.

The anatomical distribution of [18F]-T807 retention was interpreted as atypical and suggestive of the distribution of pathology in Progressive Supranuclear Palsy (PSP). That interpretation was noted in the Mitsis *et al.* (2014) paper ^1^ in light of a recent case report linking CTE and PSP ^3^ and because the subject in the Mitsis *et al.* (2014) ^1^ case manifested nasal speech, hypomimia, and impaired upgaze, all features of PSP. These facts notwithstanding, we emphasize that we cannot exclude the possibility that the pattern of [18F]-T807 binding observed in the retired NFL player could be the result of nonspecific retention of [18F]-T807 in these same regions of brain. Indeed, some as-yet unpublished experience with [18F]-T807 has demonstrated a propensity of the ligand to bind to the SN and GP in what appears to be a non-specific fashion ( http://www.alzforum.org/news/conference-coverage/tau-tracers-shine-boston-conference). The [18F]-T807 binding to the hippocampi in ^1^ could fall within the spectrum of aging-related tauopathy, the known deposition of NFT in this region ( http://www.alzforum.org/news/conference-coverage/tau-tracers-shine-boston-conference). We write here to emphasize the point that proof of the histological underpinnings of [18F]-T807 binding awaits the presentation of a sufficiently powered study of *in vivo*-radiological/postmortem-histological correlation relationships, and as a reminder that while the development of several putative tau-binding ligands has opened new possibilities for research and clinical use, that none have been validated to the extent necessary for reliable use and such validation should be a top priority.
