# Supplementary material for: [18F]-T807 tauopathy PET imaging in chronic traumatic encephalopathy
Source: F1000Res. 2014 Sep 29;3:229. [Version 1] doi: 10.12688/f1000research.5372.1 (PMC4240239; doi:10.12688/f1000research.5372.1)
Supplement: Peer review history

# Peer review: Referee response for version 1

**DOI:** 10.5256/f1000research.5735.r6684

**Published:** 2014-11-20

**Authors:** Nobuyuki Okamura

**Affiliations:** 1 Department of Pharmacology, Tohoku University, Sendai, Japan

In this letter, the authors describe an alternative explanation of [ ^18^F]T807 PET findings in patients with chronic traumatic encephalopathy (CTE). A striking article by Mitsis *et al.* (2014) has suggested the potential usefulness of tau PET imaging for the early detection of tau deposits in patients with CTE. The authors of the letter state that tracer signals in the substantia nigra and globus pallidus might be the result of nonspecific retention of [ ^18^F]T807. However, the amount of hippocampal [ ^18^F]T807 retention (SUVR 1.45) was considerably higher than the values that have been previously reported in healthy controls, suggesting the involvement of the hippocampus in this patient. It is important to confirm whether the hippocampal [ ^18^F]T807 retention was within normal age-appropriate levels or whether it reflected CTE tau lesions in this case. Furthermore, the current findings should be confirmed by postmortem examinations in the future.

I have read this submission. I believe that I have an appropriate level of expertise to confirm that it is of an acceptable scientific standard.

# Peer review: Referee response for version 1

**DOI:** 10.5256/f1000research.5735.r6252

**Published:** 2014-10-06

**Authors:** Victor Villemagne

**Affiliations:** 1 The Florey Institute of Neuroscience and Mental Health, University of Melbourne, Parkville, Australia

This timely letter by Drs Gandy and DeKosky sheds light onto the inherent difficulties of using not yet fully characterized imaging markers where lack of validation of the tracers used, coupled with limited experience in an emergent field, might potentially lead to over- or under-interpretation of the results, especially when no controls are available to juxtapose the findings. This in turn might reflect badly on the value of this new approach. Larger cohort studies and, as the authors point out, neuropathological confirmation of the imaging findings will be required to fully validate and characterize the binding of these new and essential markers for the *in vivo* imaging of tau deposits in the brain.

I have read this submission. I believe that I have an appropriate level of expertise to confirm that it is of an acceptable scientific standard.
